# Can the risk of secondary cancer induction after breast conserving therapy be reduced using intraoperative radiotherapy (IORT) with low-energy x-rays?

**DOI:** 10.1186/1748-717X-6-174

**Published:** 2011-12-16

**Authors:** Muhammad Hammad Aziz, Frank Schneider, Sven Clausen, Elena Blank, Carsten Herskind, Muhammad Afzal, Frederik Wenz

**Affiliations:** 1 Department of Radiation Oncology, University Medical Centre Mannheim, University of Heidelberg, Mannheim, Germany; 2 Department of Physics, The Islamia University of Bahawalpur, Bahawalpur, Pakistan

**Keywords:** Secondary cancer induction, radiotherapy, breast cancer, intraoperative radiotherapy, accelerated partial breast irradiation

## Abstract

**Background:**

Radiation induced secondary cancers are a rare but severe late effect after breast conserving therapy. Intraoperative radiotherapy (IORT) is increasingly used during breast conserving surgery. The purpose of this analysis was to estimate secondary cancer risks after IORT compared to other modalities of breast radiotherapy (APBI - accelerated partial breast irradiation, EBRT - external beam radiotherapy).

**Methods:**

Computer-tomography scans of an anthropomorphic phantom were acquired with an INTRABEAM IORT applicator (diameter 4 cm) in the outer quadrant of the breast and transferred via DICOM to the treatment planning system. Ipsilateral breast, contralateral breast, ipsilateral lung, contralateral lung, spine and heart were contoured. An INTRABEAM source (50 kV) was defined with the tip of the drift tube at the center of the spherical applicator. A dose of 20 Gy at 0 mm depth from the applicator surface was prescribed for IORT and 34 Gy (5 days × 2 × 3.4 Gy) at 10 mm depth for APBI. For EBRT a total dose of 50 Gy in 2 Gy fractions was planned using two tangential fields with wedges. The mean and maximal doses, DVHs and volumes receiving more than 0.1 Gy and 4 Gy of organs at risk (OAR) were calculated and compared. The life time risk for secondary cancers was estimated according to NCRP report 116.

**Results:**

IORT delivered the lowest maximal doses to contralateral breast (< 0.3 Gy), ipsilateral (1.8 Gy) and contralateral lung (< 0.3 Gy), heart (1 Gy) and spine (< 0.3 Gy). In comparison, maximal doses for APBI were 2-5 times higher. EBRT delivered a maximal dose of 10.4 Gy to the contralateral breast and 53 Gy to the ipsilateral lung. OAR volumes receiving more than 4 Gy were 0% for IORT, < 2% for APBI and up to 10% for EBRT (ipsilateral lung). The estimated risk for secondary cancer in the respective OAR is considerably lower after IORT and/or APBI as compared to EBRT.

**Conclusions:**

The calculations for maximal doses and volumes of OAR suggest that the risk of secondary cancer induction after IORT is lower than compared to APBI and EBRT.

## Background

The breast is the most common site of cancer in women and with the wide-spread use of mammography more than two-thirds of breast cancers are diagnosed at an early stage [1,2]. Early stage breast cancer carries a better prognosis, with outcomes having improved dramatically over the last two decades with a 25% reduction of breast cancer mortality [3]. Breast-conserving surgery (BCS) followed by external-beam whole-breast radiotherapy (EBRT) has become the standard of care in early breast cancer. Adjuvant EBRT after BCS significantly reduces the risk for in-breast tumor recurrence (IBTR) and improves overall survival over BCS alone [4-6].

There is clear evidence for the association between radiation exposure and cancer induction, especially from epidemiological studies of survivors of the atomic bombings in Japan [7,8]. The importance of secondary cancer risks after radiation therapy has been recognized by several international organizations, including the International Commission on Radiological Protection (ICRP), the National Council on Radiation Protection and Measurement (NCRP), and the American Association of Physicist in Medicine (AAPM). Xu et al. showed that secondary tumors occur most often in organs that are closest to radiation fields. Organs located far from the tumor volume (out-of-field organs) are assumed to receive low doses of radiation and, therefore, are frequently ignored in treatment planning, even though it is well known that small radiation doses to these organs can induce secondary cancers as well [9,10].

Radiation therapy for breast cancer has changed significantly in the last decades, regarding radiation type, application of treatment, treatment duration and 3D dose distributions. For early stage breast cancer, multiple techniques have been developed recently for accelerated partial breast irradiation (APBI) such as high dose rate brachytherapy (HDR-BT) [11,12], permanent breast seed implant (PBSI) [13], intraoperative radiotherapy (IORT) using 50 kV X-rays (TARGIT) [14] or electrons (ELIOT) [15], and 3D conformal radiotherapy [16]. All techniques share a potential advantage in patient acceptance due to higher convenience. Regardless, advances in breast radiotherapy treatment techniques should continue to focus on reducing the dose to critical structures (lung, heart, and contralateral breast) as minimal as possible to reduce the risk of secondary malignancy and of late cardiac and pulmonary complications.

IORT with low-energy X-rays (TARGIT - TARGeted Intraoperative radioTherapy) is an innovative technique that can be used during breast-conserving surgery as a sole treatment for low risk patients or as a tumor bed boost followed by external beam radiotherapy (EBRT) [14,17]. The TARGIT A study has demonstrated the non-inferiority of IORT using the TARGIT approach in selected patients as compared to standard external beam radiotherapy [14]. The purpose of the present analysis was to calculate doses to OAR and to estimate second cancer risks after IORT compared to other modalities of breast irradiation (APBI, EBRT).

## Methods

Three different breast irradiation protocols were selected which are currently used during or after breast conserving surgery in clinical practice or clinical studies. Please note that the target volume concepts and the prescription doses are different between the respective methods:

(1) IORT: single dose of 20 Gy (Intrabeam) prescribed at the applicator surface (0 mm) as used in the TARGIT A trial [14]. The target volume concept is represented by the sphere of equivalence [18].

(2) APBI: accelerated partial breast irradiation with 34 Gy in 10 fractions (5 days × 2 × 3.4 Gy) prescribed at 10 mm depth from the Intrabeam applicator surface [19] as used with Mammosite [11]. The planning target volume is 1 cm breast tissue around the excision cavity.

(3) EBRT: standard three dimensional conformal radiotherapy with 50 Gy in 25 fractions to the whole breast using two tangential fields with wedges. Here, the planning target volume is considerably larger and includes the whole breast including a safety margin.

**Figure 1:**
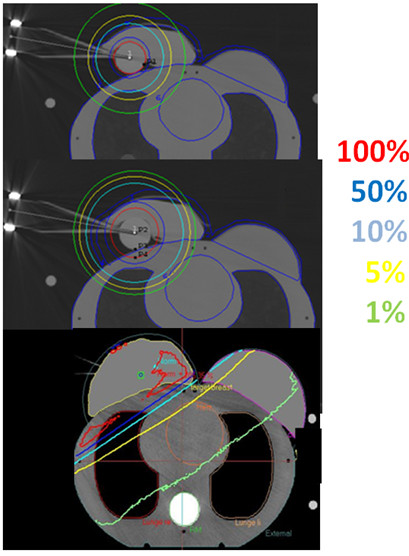
**Planning CT of an anthropomorphic phantom with an Intrabeam applicator in the upper outer quadrant of the right breast showing calculated isodoses (1%-100%)**. (a) IORT (20 Gy at 0 mm, 50 kV). (b) APBI (34 Gy at 10 mm, 50 kV). (c) EBRT (50 Gy, 6 MV).

CT scans were obtained from a CT-simulator (Brilliance CT Big Bore, Philips, Cleveland, OH, USA) of an anthropomorphic phantom (Model: 002LFC, CIRS, USA) having a single breast attachment as shown in Figure 1. An Intrabeam applicator (4 cm diameter) (Carl Zeiss Surgical, Oberkochen, Germany) was inserted in the upper outer quadrant of the breast.

All images were transferred via DICOM to the treatment planning system (Nucletron Plato Brachytherapy planning system, version 14.2.6, Veenendaal, The Netherlands). The organs at risks (OARs) such as ipsilateral breast, contralateral breast, ipsilateral lung, contralateral lung, spine and heart were defined and contoured accurately.

For isodose distribution of IORT and APBI, we used the spherical Intrabeam applicator with 4 cm in diameter. We defined an Intrabeam source (50 kV) in the treatment planning system and placed the source at the center of the spherical applicator. By using the CT images of the breast phantom, only one source-dwell position was planned to deliver a dose of 20 Gy at 0 mm from the applicator surface for IORT and 34 Gy in 10 fractions (5 days × 2 × 3.4 Gy) at 10 mm depth from the applicator surface for APBI (see Figure 1). The isodose lines of 1%, 5%, 10%, 50%, 100% were selected by using the dose preferences option in Plato for IORT and APBI as shown in figures 1 and 1. The 3D dose distribution was then calculated and stored for evaluation purposes.

For conventional EBRT, all planning CT images were transferred via DICOM to Oncentra Masterplan (version 3.3, Nucletron, Sweden) for the 3D dose calculation. The OARs were contoured in the Oncentra Masterplan software in the same way as for IORT and APBI in the Plato brachytherapy planning software. Using CT based 3D treatment planning; a total dose of 50 Gy in 2 Gy fractions was prescribed to the whole ipsilateral breast using standard tangential treatment portals (6 MV, Synergy, Elekta, Crawley/UK) as shown in figure 1. Each field included a wedge to achieve a homogeneous dose distribution inside the breast volume. For the comparison of IORT, APBI and EBRT, we selected the same isodose lines of 1%, 5%, 10%, 50%, 100%.

After calculation of the dose distribution, Dose-Volume Histograms (DVHs) were calculated for all OARs for complete evaluation of the treatment plan for all three techniques (IORT vs. APBI vs. EBRT). The mean and maximum doses and volumes receiving more than 0.1 Gy and 4 Gy of the OARs were calculated and compared. Some values such as 0.1 Gy to 0.3 Gy for IORT and 0.1 Gy to 0.5 Gy for APBI were calculated by option polynomial with the standard Software Excel™ (Microsoft Corporation, Redmond, Seattle, USA), because in Plato brachytherapy system, it was not possible to calculate values for lower doses and also volumes according to these doses.

The lifetime probabilities of developing fatal secondary malignancies were calculated per Sv absorbed in breast and lung using the National Council on Radiation Protection and Measurements (NCRP) report 116 Table Seven Part Two page 32 [20], according to a similar study by Pignol et al. [21].

## Results

Figure 1 shows the comparison of the isodose distributions from the three breast radiotherapy techniques. Obviously there are large differences in the dose distributions and especially in the low doses regions delivered to the OARs. Compared with IORT, both APBI and EBRT, deliver higher doses to the ipsilateral lung and heart. Both IORT and APBI deliver negligible doses to the spine, contralateral lung and contralateral breast as compared to EBRT.

**Figure 2:**
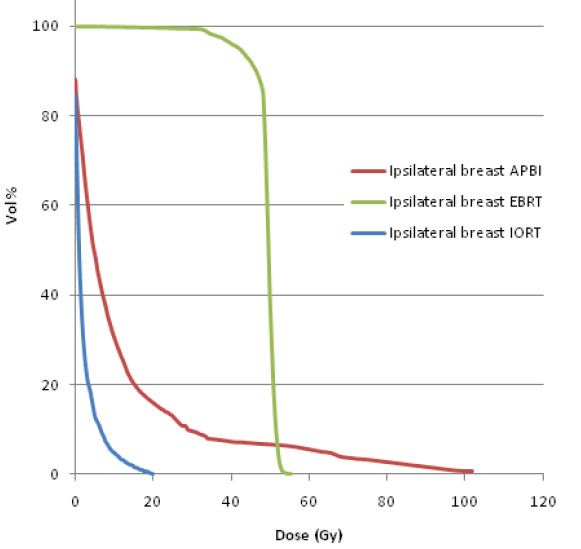
**Cumulative DVH for ipsilateral breast for IORT, APBI and EBRT**.

**Figure 3:**
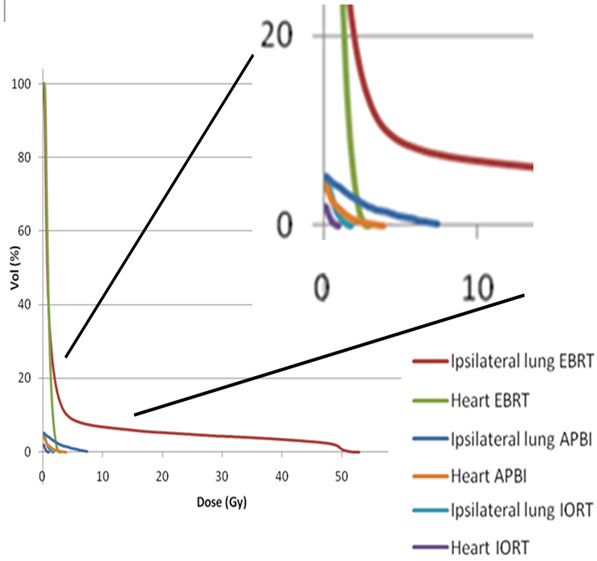
**Cumulative DVH for ipsilateral lung and heart for IORT, APBI and EBRT**.

DVHs provide dose volume information for the organs contoured in the treatment planning system. Figure 2 shows the dose volume contribution to the ipsilateral breast from the different breast radiotherapy techniques. Most of the volume of the ipsilateral breast receives almost 100% dose by EBRT. In contrast, a marked dose reduction to large volumes is seen in the ipsilateral breast by the partial breast irradiation techniques IORT and APBI. However, due to the steep dose gradient and the prescription to 10 mm tissue depth, APBI delivers the highest maximal dose to the ipsilateral breast. Figure 3 shows the comparison of DVHs of ipsilateral lung and heart by IORT, APBI and EBRT. Ipsilateral lung and heart receive considerably higher doses by EBRT as compared to IORT. Interestingly, APBI results in a higher maximal dose to the heart compared to EBRT even for a case with right breast cancer. The doses to relevant volumes of critical structures like ipsilateral lung and heart are minimal and can be considered negligible by IORT.

To estimate the risk of stochastic and deterministic normal tissue effects, table 1 compares the mean and maximal doses to selected organs during IORT, APBI and EBRT. Mean and maximal doses in the OARs delivered by IORT are consistently lower as compared to APBI and EBRT. The maximal dose to the heart is larger during APBI than after EBRT and considerably smaller using IORT. The external beam radiotherapy yields the largest maximal dose in the ipsilateral lung (53 Gy), contralateral lung (1.0 Gy) and contralateral breast (10.4 Gy). Due to very low mean doses in the spine, contralateral lung and contralateral breast after IORT and APBI with less than 1.5% of the prescribed dose, it was not possible to calculate exact values for these OARs with the Plato brachytherapy system.

**Table 1:** Maximal and mean doses for OARs for IORT, APBI and EBRT.

Dose to Organs	IORT		APBI		EBRT	
	**Mean dose (Gy)**	**Max dose (Gy)**	**Mean dose (Gy)**	**Max dose (Gy)**	**Mean dose (Gy)**	**Max dose (Gy)**

**Ipsilateral Breast**	2.2	20	10.4	102	49.0	55.3

**Contralateral breast**	**......**.	< 0.3	**.......**.	< 0.56	1.1	10.4

**Ipsilateral Lung**	0.03	1.8	0.13	7.4	3.4	53.0

**Contralateral lung**	**.....**.	< 0.3	**.......**.	< 0.56	0.24	1.0

**Heart**	0.01	1	0.06	3.8	1	2.8

**Spine**	**......**.	< 0.3	**.......**.	< 0.56	0.24	0.5

Doses < 1.5% of the prescribed dose cannot be shown by Plato (......).

As an estimate for secondary cancer risk, table 2 compares the corresponding volumes of OARs receiving more than 0.1 Gy and 4 Gy from the different breast radiotherapy protocols. The ipsilateral breast shows a smaller volume for doses higher than 0.1 Gy and 4 Gy after IORT than in the case of APBI and EBRT. There is a larger volume with doses > 4 Gy within the ipsilateral lung for EBRT than after APBI, while this dose is not reached by IORT at all. For external beam radiotherapy, almost the total volume for spine, contralateral lung and contralateral breast receive doses of > 0.1 Gy while these organs show negligible volumes receiving more than 0.1 Gy by IORT and APBI. With the EBRT technique, about 10% of the contralateral breast receives doses > 4 Gy. There is no volume for heart, spine and contralateral lung receiving doses higher than 4 Gy from these radiotherapy protocols (IORT vs. APBI vs. EBRT).

**Table 2:** Volumes of OAR receiving doses greater than 0.1 Gy and 4 Gy which are doses considered to be relevant for induction of secondary cancers.

	IORT		APBI		EBRT	
	**%Vol > 0.1 Gy**	**%Vol > 4 Gy**	**%Vol > 0.1 Gy**	**%Vol > 4 Gy**	**%Vol > 0.1 Gy**	**%Vol > 4 Gy**

**Ipsilateral Breast**	84.5	18.1	88.2	54.4	99.9	99.9

**Contralateral breast**	< 1	0	< 1	0	97.7	1.3

**Ipsilateral Lung**	4.5	0	5.0	1.3	98.8	10.1

**Contralateral lung**	< 1	0	< 1	0	87	0

**Heart**	1.8	0	4.2	0	98	0

**Spine**	< 1	0	< 1	0	92	0

Table 3 compares the secondary cancer risk estimates from these breast radiotherapy techniques. We calculated the secondary cancer risk from maximal doses where mean doses were not available but obviously then this can only be an upper estimation of the risk. In the case of ipsilateral lung, secondary cancer risk (0.02%) is considerably less for IORT as compared to APBI and EBRT. The secondary cancer risk for contralateral breast (< 0.06%) calculated from maximal dose for IORT is lower than for APBI and EBRT. The calculated risk from EBRT for the ipsilateral and contralateral lung is about 2.9% and 0.2%.

**Table 3:** Lifetime risk of secondary cancers for organs doses from different breast radiotherapy techniques.

Organs	Probability (%/Sv)	IORT	APBI	EBRT
**Contralateral Breast**	0.20	(< 0.06%*)	(< 0.11%*)	0.22% (< 2.08%*)

**Ipsilateral lung**	0.85	0.02%	0.11%	2.9%

**Contralateral lung**	0.85	(< 0.25%*)	(< 0.47%*)	0.2% (< 0.85%*)

The probability per Sievert was taken from the National Council on Radiation Protection and Measurements (NCRP) Report 116. Mean organ doses were used for the calculation of secondary cancer risk (*maximal doses were used where mean doses were not available). Note that RBE effects were not taken into account, which may be up to a factor of 1.3 - 3 for IORT (see discussion).

## Discussion

Over the past several years, multiple studies have established the feasibility of IORT for the treatment of selected early-stage breast cancer patients. The goal of the intraoperative approach is to deliver a high dose to parts of the breast, i.e. the tissue around the tumor cavity up to a depth of 1-2 cm, within a short overall treatment time (APBI - accelerated partial breast irradiation). In contrast to other approaches, where the dose is prescribed to a conventional PTV margin, in the TARGIT approach [14,17] using a low-energy X-ray device (Intrabeam), the dose is not prescribed to a defined depth. The highest dose is at the applicator surface and it decreases with increasing tissue distance from the applicator. This steep dose fall-off results in very low doses to surrounding organs and eliminates the need for specific radiation protection measures. Reports about local tumor control and acute and long-term side effects have been published with follow-up times up to ten years. It is obvious, that there are no clinical analyses about second cancer induction using this approach due to the limited time span of clinical availability. To estimate the long term risks of breast radiotherapy including secondary cancer, we performed dosimetric comparisons of IORT using Intrabeam with selected other breast radiotherapy techniques.

High-dose radiation increases the risk of second malignancy after breast or chest-wall irradiation (e.g. Surveillance Epidemiology and End Results (SEER) database) [22] (see table 4 for overview). Hall et al. showed that the risk increases with a linear proportionality to dose [23,24] between low doses and moderate doses (from 0.1 Gy to 3 Gy). Little [25] distinguished the difference between A-bomb survivors and patients treated by radiotherapy to the role of cell killing at doses higher than 2 Gy but the analysis of Le Pogam et al. [26] did not provide evidence for a role of cell killing. Other reports suggested a threshold at about 0.6 Gy in adults after fractionated radiotherapy and after an acute irradiation in children at 0.1 Gy [26,27]. We therefore chose volumes receiving more than 0.1 Gy (threshold) and 4 Gy (relevant dose) for comparison in our analysis.

**Table 4:** Secondary cancer risk after radiotherapy for breast cancer

Authors, journal, year	No. of patients	Follow up time	organs	Risk for secondary cancer
Berrington de Gonzalez Br J Cancer (2010) [22]	182,057 69,181 treated with radiotherapy	13 years	Lung, oesophagus, pleura, bone and soft tissue Contralateral breast	RR = 1·38 (95% CI 1·26-1·51) RR = 1.09 (95% CI 1.04-1.15)

Kirova Int J Radiat Oncol Biol Phys (2007) [53]	16,705 13,472 treated with radiotherapy	10 years	Lung Contralateral breast	RR = 3.09 (95% CI 1.12-8.53) RR = 1.1 (95% CI 0.96-1.27)

Roychoudhuri Br J Cancer (2004) [54]	64,782 33,763 received radiotherapy	> 10 years	Lung	RR = 1.65 (95% CI 1.05-2.54)

Zablotska Cancer (2003) [31]	260,541 76,467 treated with radiotherapy	10 years	lung	0.30% RR = 2.06 (95% CI 1.53-2.78)

Prochazka Eur J Cancer (2002) [55]	141,053 141,053 treated with radiotherapy	> 10 years	lung	RR = 2.3 (95% CI 1.97-2.63)

Fowble Int J Radiat Oncol Biol Phys (2001) [28]	1,253 977 treated with radiotherapy	10 years	Contralateral breast	7%

Obedian J Clin Oncol (2000) [29]	2,416 1,029 treated with radiotherapy	15 years	Contralateral breast	10%

There is a wide variation of risk estimates for second cancer in the literature. Lifetime breast cancer induction risk for a breast exposed to 1 Gy is approximately 5% if irradiated at the age of < 35, < 3% at the age of 35-45, and much less at an older age [10]. For a phantom case study [21] the incremental risk of secondary cancer was calculated for the tangential whole breast technique with wedge compensators based on National Council on Radiation Protection and Measurements (NCRP) report 116 [20] as 0.34% which is likely to be undetectable compared to the observed frequency of contralateral breast cancer of about 7% at 10 years and 10% at 15 years [28,29]. The causes of contralateral breast cancer amongst breast cancer patients given radiotherapy are less obvious. A large study by Kirova et al. did not show an increased risk of contralateral breast cancer for those receiving radiotherapy [30]. Obedian reported no significant difference in the occurrence of contralateral breast cancer at 15 years in a retrospective series of 2,416 patients treated with breast conserving surgery and adjuvant radiotherapy or mastectomy without radiotherapy [29].

The calculated risk for lung cancer after EBRT in a phantom study was 0.49% [21]. This value was slightly higher but of the same order of magnitude than the 0.30% increased risk for adjuvant radiotherapy found by Zablotska on a cohort of 260,000 patients included in the Surveillance Epidemiology and End Results (SEER) database [31]. For the particular case of the irradiation of the lung during the treatment for breast cancer, Inskip et al. [32] have concluded that for an average dose of 10 Gy the risk for radiation-induced secondary cancer is around 0.9% which represents about a twofold increase of risk of pulmonary neoplasia among 10-year survivors of breast cancer.

The calculated doses to the OAR in our study are considerably lower with IORT as compared to standard tangential EBRT and therefore the estimated risk for secondary cancer should be considerably lower. However, one may assume that novel EBRT approaches using tangential intensity modulated radiation therapy (IMRT) replacing wedges may yield lower scattered doses in the range of a factor of 2. In contrast, rotational IMRT techniques or isotropic multi-field IMRT may be associated with a dose bath, i.e. a large volume receiving low doses [24]. Intensity-modulated radiotherapy has been developed to improve the homogeneity of the dose distribution within the target volume, but, by contrast with 3D radiotherapy, is generally associated with a larger volume of healthy tissue being irradiated to low doses. This is due to an increase in the number of beams used with this technique and the number of monitor units, resulting in radiation leakage, and an increase in the total body exposure. These two factors could lead to an increase in the risk of second cancers [23,24]. A study by Lettmaier et al. showed that maximum doses received by different volumes of the heart, the lungs and the skin, and dose values for all OAR are consistently lower for partial breast irradiation using brachytherapy than those for whole breast irradiation with EBRT [33].

Another important point for comparative risk estimation is that low energy X-rays have an increased relative biological effectiveness (RBE). The maximal doses to OARs in our study are 3 - 20 times lower after IORT as compared to EBRT. RBE values of 1.3 up to 3 have been reported for Intrabeam [18,19,34] which would still result in lower maximal biological doses as compared to EBRT. Additional uncertainty of the risk estimation is based on the different dose rate and time schedule i.e. fractionated application over 6 weeks vs. single dose.

The estimation of secondary cancer risks after breast cancer therapy is also complicated by the fact that not only radiotherapy may be associated with mutagenesis. Several studies have reported an increased risk for myeloid leukemia (AML) or myelodplastic syndrome (MDS) in breast cancer patients treated with adjuvant chemotherapy. Praga et al. [35] reviewed 7110 patients treated with epirubicin and cyclophosphamide in 19 randomized clinical trials. At a median follow-up of 8 years, the cumulative probability of AML or MDS was 0.55%. The risk increased in relation to the cumulative doses of both agents. Patients who received cumulative doses not exceeding those used in standard regimens (720 and 6300 mg/m^2^, for epirubicin and cyclophosphamide, respectively) had an 8-year probability of 0.37% (95% confidence interval (CI) 0.13% to 0.61%) compared with 4.97% (95% CI 2.06% to 7.87%)) for those who received higher doses [35,36]. Furthermore, oral bisphosphonates have been associated with an increased risk of esophageal cancer. Green and colleagues, who used the UK's General Practice Research Database compared the frequency of oral bisphosphonate exposure in cases versus matched non-cases. They found that one (or more) prescription for oral bisphosphonates increased the risk of esophageal cancer by 30%; 10 or more prescriptions nearly doubled the risk. They found a globally increased risk (Relative Risk (RR): 1.3; 95% Confidence Interval (95% CI): 1.02-1.66), and an almost doubled risk in patients who used at least 10 prescriptions (RR = 1.93; 95% CI 1.37-2.70) or in those who were treated for more than 3 years (RR: 2.24; 95% CI: 1.47-3.43) [37,38].

Radiation induced late heart disease has been observed in patients who received therapeutic doses of about ≥ 35 Gy to partial volumes of the heart [39]. Recent studies based on atomic bomb survivors also suggest a relationship between cardiac mortality and low radiation doses in the range of ≤ 4 Gy [7,40-42]. Several clinical case series found no clear evidence of late cardiac mortality after breast radiotherapy [43-46]. In contrast, a recent critical view published by Schultz-Hector suggests that acute single doses of 1-2 Gy to the heart increased the risk of developing ischemic heart disease significantly [47]. Considering this, it is of interest to notice that APBI delivers a higher maximal dose to parts of the heart as compared to IORT and EBRT. A significantly better sparing of the high-dose volume of the heart in selected early breast cancer patients with unfavourable thoracic geometry has been reported by the use of multifield IMRT [48-50]. Compared to three dimensional conformal radiotherapy (3DCRT), multifield IMRT reduced the heart volume receiving ≥ 30 Gy by 87% [48], or ≥ 35 Gy by 81% [49].

Modelling cardiac toxicity is complicated for several reasons, as discussed in the review by Gagliardi et al. [51], including organ at risk definition (heart or left ventricle or myocardium), scarce dosimetric data for historical techniques, and a latency of symptoms of > 10 years. Although the mean excess NTCP (cardiac mortality) for tangent RT in their series of 100 consecutive patients was about 2%, they reported a subset of patients with an excess NTCP of 4-5%. In a previous report from our group we worked with a cohort of patients selected because of their unfavorable anatomy, which is probably comparable to their group with the greatest NTCP. Using multifield IMRT a similar order of magnitude of NTCP reduction (3-4% to 0.05% for their sample patient and 6.03% to 0.25% for our patients) could be achieved [48,50,52].

## Conclusions

This is to our knowledge the first report about the estimation of second cancer risk using TARGIT IORT (50 kV X-rays) for breast cancer. In comparison with APBI and EBRT, the calculated mean and maximal doses for OAR are lower for IORT, as well as the high dose volumes (> 4 Gy). This would suggest that the risk of secondary cancer induction after IORT is lower than after APBI or EBRT.

## List of abbrevations

(APBI): Accelerated partial breast irradiation; (AAPM): American Association of Physicist in Medicine; (BCS):Breast-conserving surgery; (CI): Confidence interval; (ELIOT): Electron intraoperative therapy; (EBRT): External beam radiotherapy): DVHs): Dose volume Histograms; (HDR-BT): High dose rate brachytherapy; (IBTR): In-breast tumor recurrence; (IMRT): Intensity modulated radiation therapy; (ICRP): International Commission on Radiological Protection; (IORT): Intraoperative radiotherapy; (MDS): Myelodplastic syndrome; (AML): Myeloid leukemia; (NCRP): National Council on Radiation Protection and Measurement; (NTCP): Normal tissue complication probability; (OAR): Organ at risks; (PBSI): Permanent breast seed implant; (PTV): Planning target volume; (RT):Radiotherapy; (RBE): Relative biological effectiveness; (RR): Relative Risk; (SEER): Surveillance epidemiology and end results; (TARGIT): TARGeted Intraoperative radiotherapy; (3DCRT): Three dimensional conformal radiotherapy; (3D): Three dimensional.

## Competing interests

Carl Zeiss Surgical supports radiobiological research at Department of Radiation and Oncology, University Medical Center Mannheim, University of Heidelberg, Mannheim, Germany.

## Authors' contributions

MHA, MA and FW were responsible for the concept and design of the project. MHA, FS, SC and EB performed the phantom experiments. FS, SC and MA were responsible for the dosimetric calculations. CH performed the estimations of cancer risk and was responsible for the radiobiological aspects. MHA, EB, CH and FW drafted the manuscript. MHA, FS, SC, EB, CH, MA and FW performed proofreading and final corrections of the manuscript. All authors have read and approved the final version of the manuscript.

## Authors' information

Department of Radiation and Oncology, University Medical Center Mannheim, University of Heidelberg, Germany
